# The biomarker HE4 (WFDC2) promotes a pro-angiogenic and immunosuppressive tumor microenvironment via regulation of STAT3 target genes

**DOI:** 10.1038/s41598-020-65353-x

**Published:** 2020-05-22

**Authors:** Nicole E. James, Jenna B. Emerson, Ashley D. Borgstadt, Lindsey Beffa, Matthew T. Oliver, Virginia Hovanesian, Anze Urh, Rakesh K. Singh, Rachael Rowswell-Turner, Paul A. DiSilvestro, Joyce Ou, Richard G. Moore, Jennifer R. Ribeiro

**Affiliations:** 1 Women and Infants Hospital, Department of Obstetrics and Gynecology, Program in Women’s Oncology, Providence, RI USA; 2 Warren-Alpert Medical School of Brown University, Providence, RI USA; 3 Rhode Island Hospital, Digital Imaging and Analysis Core Facility, Providence, RI USA; 4 Northwell Health Physician Partners Gynecologic Oncology, Brightwaters, NY USA; 5 University of Rochester Medical Center, Rochester, NY USA; 6 Women and Infants Hospital, Department of Pathology, Providence, RI USA

**Keywords:** Cancer microenvironment, Ovarian cancer, Tumour angiogenesis

## Abstract

Epithelial ovarian cancer (EOC) is a highly lethal gynecologic malignancy arising from the fallopian tubes that has a high rate of chemoresistant recurrence and low five-year survival rate. The ovarian cancer biomarker HE4 is known to promote proliferation, metastasis, chemoresistance, and suppression of cytotoxic lymphocytes. In this study, we sought to examine the effects of HE4 on signaling within diverse cell types that compose the tumor microenvironment. HE4 was found to activate STAT3 signaling and promote upregulation of the pro-angiogenic STAT3 target genes IL8 and HIF1A in immune cells, ovarian cancer cells, and endothelial cells. Moreover, HE4 promoted increases in tube formation in an *in vitro* model of angiogenesis, which was also dependent upon STAT3 signaling. Clinically, HE4 and IL8 levels positively correlated in ovarian cancer patient tissue. Furthermore, HE4 serum levels correlated with microvascular density in EOC tissue and inversely correlated with cytotoxic T cell infiltration, suggesting that HE4 may cause deregulated blood vessel formation and suppress proper T cell trafficking in tumors. Collectively, this study shows for the first time that HE4 has the ability to affect signaling events and gene expression in multiple cell types of the tumor microenvironment, which could contribute to angiogenesis and altered immunogenic responses in ovarian cancer.

## Introduction

Epithelial ovarian cancer (EOC) is a highly lethal gynecologic malignancy arising from the fallopian tubes^1,2^. It is frequently diagnosed at an advanced stage, and many patients develop a recurrence within 12–18 months of finishing their primary treatment regimen of traditional platinum based-chemotherapeutics^3^. The five-year survival rate for EOC in the United States is only 47%^4^, necessitating an emphasis on the development of novel targeted agents. The introduction of anti-angiogenic therapy and PARP inhibitors have led to improvements in progression free survival, but significant challenges still remain in producing long-term benefits^5–8^. Currently, EOC clinical trials center upon monoclonal antibodies against the immune checkpoint inhibitor programmed cell death-1 (PD-1), which suppresses the anti-tumor function of CD8 + T cells^9^. In EOC, PD-1 inhibitors have not exhibited the same efficacy as they have in other cancers^10^. Therefore, optimization of current immunotherapies or development of novel therapeutics is still needed in order to improve survival for EOC patients.

Anti-angiogenic, vascular endothelial growth factor (VEGF) targeted therapies enhance response to immunotherapy checkpoint blockade by promoting trafficking and activation of tumor infiltrating lymphocytes (TILs) and migration and recognition of antigen presenting cells^11–14^. This effect occurs due to the inherent interconnectedness between angiogenesis and the immune microenvironment. Hypoxia and dysfunctional tumor blood vessel formation impairs trafficking of cytotoxic CD8 + TILs and other immune cells important for anti-tumor responses, while selectively recruiting immune suppressive tumor-associated macrophages and T regulatory cells^15^. Furthermore, angiogenic factors downregulate vascular endothelium expression of cell adhesion molecules that normally interact with and directly affect various immune cell populations to promote anti-tumor immunity^16^. Likewise, immune cells secrete cytokines that impact the tumor vasculature, creating a bidirectional relationship between angiogenesis and immune suppression^17^.

Human epididymis protein 4 (HE4) is a small, secretory protein and member of the whey acidic protein (WAP) domain family with a proposed role as an antiprotease^18,19^. HE4 is an established EOC clinical biomarker that can be readily detected in patient serum and is overexpressed in EOC tissue^20,21^. *In vitro* and *in vivo* studies have also demonstrated its role in EOC tumorigenesis, chemoresistance, and metastasis^22–31^. Our recent studies were also the first to demonstrate that HE4 suppresses the cytotoxic function of peripheral blood mononuclear cells against ovarian cancer cells^32,33^. The objective of this current study was to determine the effect of HE4 on gene expression in immune cells. These studies led to the discovery of a role for HE4 in regulating angiogenesis and associated signaling pathways in cells of the tumor microenvironment, as well as a clinical association between HE4 and microvascular density and T cell numbers in patient tissue.

## Results

### HE4 regulates immune-related gene expression in peripheral blood mononuclear cells

**Figure 1:**
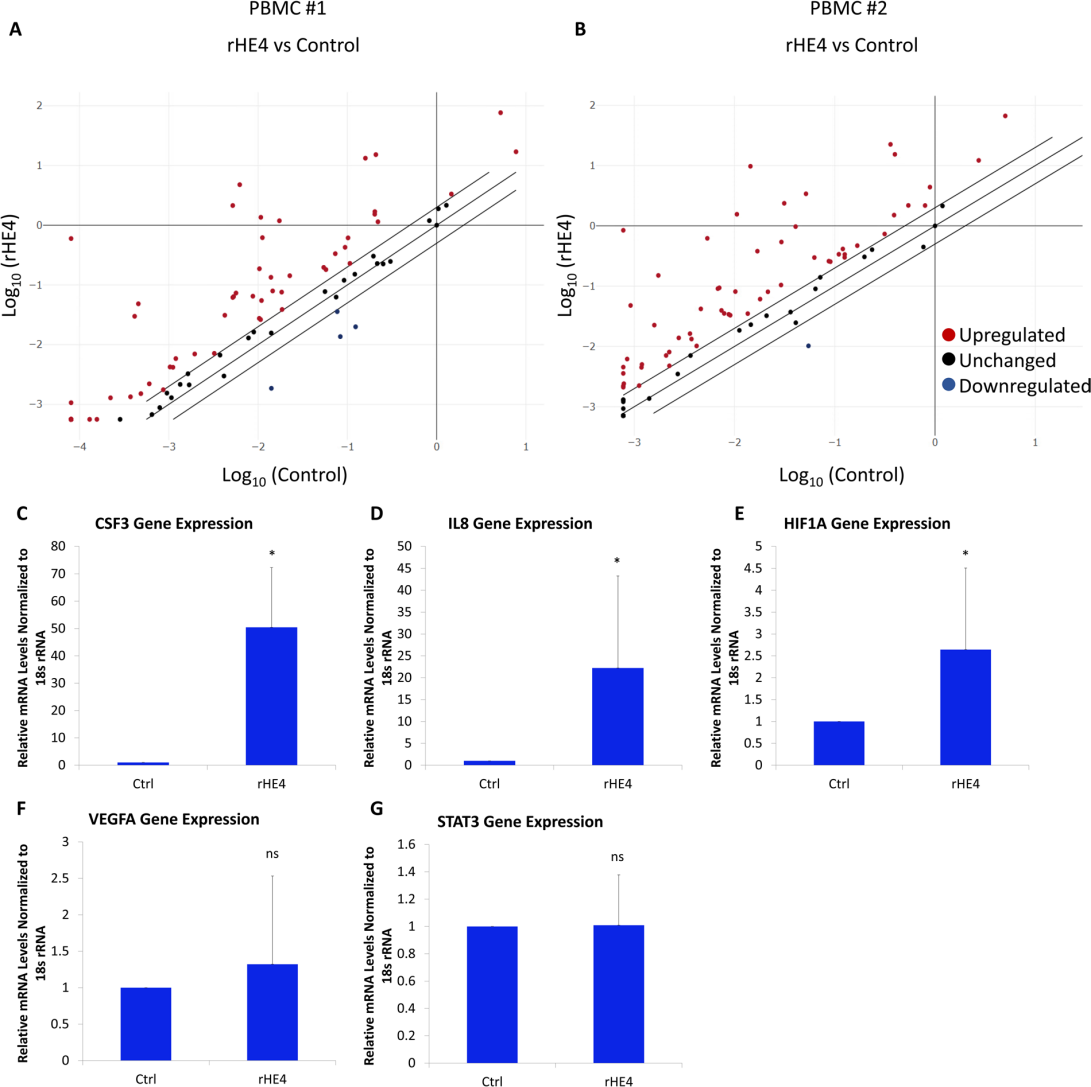
HE4 regulates immune-related gene expression in peripheral blood mononuclear cells (**A,B**) Scatter plots of gene expression determined by quantitative PCR array in control or rHE4-treated PBMC. qPCR was performed to validate genes changes, revealing upregulation of *CSF3*, *IL8*, and *HIF1A* with rHE4 treatment of PBMC **(C–E)**. No change in *STAT3* or *VEGFA* levels were observed with rHE4 treatment **(F,G)**. Error bars represent standard deviation. *p < 0.05. Results are the average of at least three biological replicates.

To investigate the potential effects of HE4 on immune cells, we treated two sets of normal human peripheral blood mononuclear cells (PBMC) in triplicate with 20 nM recombinant HE4 (rHE4) for 6 h. The control and rHE4-treated triplicates were pooled and quantitative PCR (qPCR) arrays (RT2 Profiler Cancer Inflammation and Immunity Crosstalk human array) were performed  to determine gene expression changes in response to treatment. There was a high degree of correlation between the results of the two sets of arrays (Pearson r = 0.8884, p < 0.0001), and all gene changes were consistent between arrays except four genes. The genes changed at least 3-fold in either direction with rHE4 treatment are listed in Table 1. A majority of genes changed were in the positive direction (Fig. 1A,B), which is consistent with the predominantly stimulatory effect we have previously noted with rHE4 treatment or overexpression.

**Table 1:** List of genes regulated by rHE4 at least 3-fold in either direction.

List of genes regulated +/−3-fold by rHE4 treatment of PBMC		
*Gene Symbol*	*Description*	*Fold-change*
CSF3	Colony stimulating factor 3	4267.63
IL6	Interleukin 6	722.035
CCL20	Chemokine (C-C motif) ligand 20	243.535
IL1A	Interleukin 1A	137.055
CXCL1	C-X-C motif chemokine ligand 1	85.695
CSF2	Colony stimulating factor 2	79.36
CCL18	Chemokine (C-C motif) ligand 18	79.05
CCL4	Chemokine (C-C motif) ligand 4	67.845
IL1B	Interleukin 1B	61.05
PTGS2	Prostaglandin-endoperoxidase synthase 2	46.3
CXCL2	C-X-C motif chemokine ligand 2	37.135
IL10	Interleukin 10	15.67
CXCL8	Interleukin 8	14.02
CXCL5	C-X-C motif chemokine ligand 5	13.595
CCR7	C-C chemokine receptor type 7	12.815
TNF	Tumor necrosis factor	12.355
IDO1	Indoleamine 2,3-dioxygenase	11.5
CD274	Programmed cell death ligand 1	10.48
CCL22	Chemokine (C-C motif) ligand 22	8.66
IFNG	Interferon gamma	7.695
CCL5	Chemokine (C-C motif) ligand 5	5.715
IL23A	Interleukin 23A	5.46
MYC	Myc proto-oncogene	5.11
GZMA	Granzyme A	4.75
GZMB	Granzyme B	4.56
HLA-B	Major histocompatibility complex, class 1, B	4.55
PDCD1	Programmed cell death 1	4.425
SPP1	Secreted phosphoprotein 1	4.25
FOXP3	Forkhead box P3	4.155
HIF1A	Hypoxia inducible factor 1A	3.99
CCL2	Chemokine (C-C motif) ligand 2	3.795
IL15	Interleukin 15	3.76
NFKB1	Nuclear factor kappa beta 1	3.615
CTLA4	Cytotoxic T-lymphocyte-associated protein 4	3.575
CCR4	C-C chemokine receptor type 4	3.5
CXCR3	C-X-C motif chemokine receptor 3	3.385
MIF	Macrophage migration inhibitory factor	3.3
FASLG	Fas ligand	3.155
IL2	Interleukin 2	3.15
CCR1	C-C chemokine receptor type 1	−5.73

While several genes, particularly colony stimulating factor 3 (*CSF3*), were highly upregulated, we were interested in the upregulation of *CXCL8* (*IL8*; interleukin 8) and hypoxia inducible factor alpha (*HIF1A*), since these genes are both involved in promoting angiogenesis and are STAT3 regulated^34,35^. Previous unpublished data indicated that HE4 promotes STAT3 activation, which we postulated could be responsible for the upregulation of at least these two genes. Furthermore, the upregulation of *IL8* was in agreement with our previously published microarray results showing *IL8* to be a top upregulated gene by rHE4 treatment of OVCAR8 cells^30^. The complete results from the qPCR array can be seen in the Supplemental Data File.

In order to validate results from the array, we treated normal human PBMC with 20 nM rHE4 and performed quantitative PCR (qPCR). We looked at expression of *CSF3* (as the most upregulated gene), *IL8*, *HIF1A*, as well as *STAT3* and *VEGFA*, which were relatively unchanged in both arrays. All gene expression levels were validated by qPCR (Fig. 1C–G).

### HE4-mediated upregulation of IL8 and HIF1A gene expression is suppressed by STAT3 inhibition in PBMC

**Figure 2:**
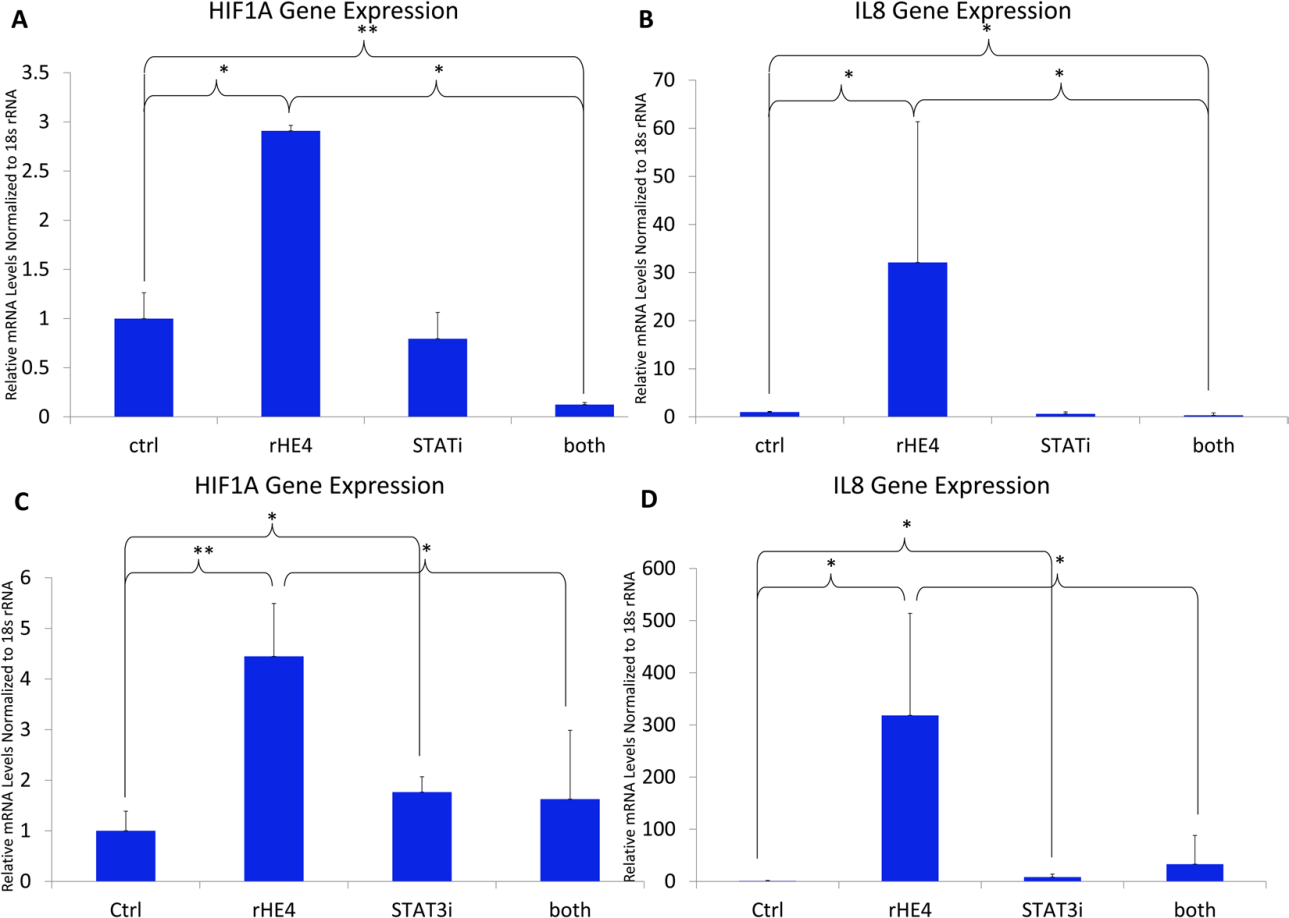
HE4-mediated upregulation of *IL8* and *HIF1A* gene expression is suppressed by STAT3 inhibition in PBMC (**A**) qPCR revealed upregulation of *HIF1A* in PBMC treated with rHE4 for 6 h, which was blocked by treatment with a STAT3 inhibitor. **(B)** Upregulation of *IL8* in PBMC treated with rHE4 for 6 h, which was blocked by treatment with a STAT3 inhibitor. **(C)** Upregulation of *HIF1A* in PBMC treated with rHE4 for 24 h, which was blocked by treatment with a STAT3 inhibitor. **(D)** Upregulation of *IL8* in PBMC treated with rHE4 for 24 h, which was blocked by treatment with a STAT3 inhibitor. Error bars represent standard deviation. *p < 0.05. Results are the average of ≥3 biological replicates.

To determine the dependency of HE4-mediated *IL8* and *HIF1A* upregulation on STAT3 signaling, we treated PBMC with 20 nM rHE4 alone or with 50 µM of an inhibitor of STAT3 (STAT3 inhibitor VIII), for 6 h. We confirmed upregulation of *IL8* (32.1-fold, p = 0.039) and *HIF1A* (2.9-fold, p = 0.010) with rHE4 treatment in these cells. Importantly, the upregulation of both *IL8* and *HIF1A* was suppressed by STAT3 inhibition (p = 0.037 and p = 0.030, respectively), suggesting that the upregulation of these two genes by HE4 is mediated by activated STAT3. (Fig. 2A,B). To examine the time-dependent nature of this effect, we treated PBMC with 20 nM rHE4 alone and with 25 µM STAT3 inhibitor for 24 h, and found that at this time point, *IL8* and *HIF1A* were further upregulated by HE4, which was again blocked by STAT3 inhibition (Fig. 2C,D).

### HE4-mediated STAT3 activation and upregulation of IL8 and HIF1A is blocked by STAT3 inhibition

**Figure 3:**
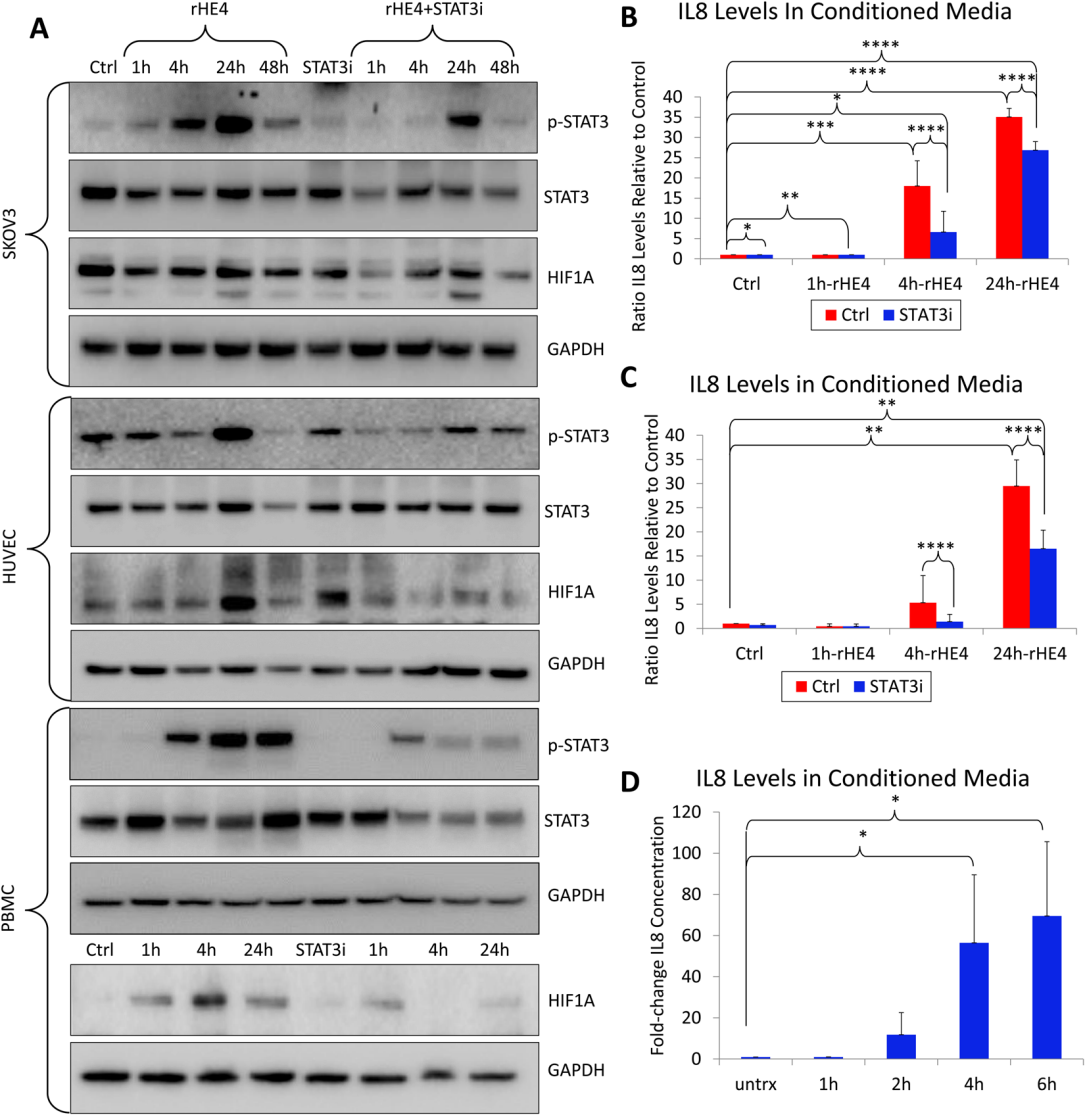
HE4-mediated STAT3 activation and upregulation of IL8 and HIF1A is blocked by STAT3 inhibition (**A**) Upregulation of phospho-STAT3 in SKOV3 cells, HUVECs, and PBMCs by rHE4 treatment. Upregulation of HIF1α by rHE4 observed in HUVECs and PBMC. STAT3 activation and HIF1A levels were ablated by treatment with a STAT3 inhibitor. Boxes separate images from the same gel for each cell type. Blots were either subsequently reprobed for various antibodies, or stripped and reprobed in the case of STAT3/p-STAT3. **(B)** ELISA revealed increased secretion of IL8 by PBMC treated with 20 nM rHE4 from 4–24 h post-treatment, which was suppressed by STAT3 inhibition. **(C)** ELISA revealed increased secretion of IL8 by PBMC treated with 1 nM rHE4 from 4–24 h post-treatment, which was suppressed by STAT3 inhibition. **(D)** ELISA revealed IL8 secretion by PBMC was increased as early as 2 h post-treatment with rHE4. Error bars represent standard deviation of ≥3 biological replicates. *p < 0.05, **p < 0.005, ***p < 0.005, ****p < 0.0005.

We next confirmed that 20 nM rHE4 treatment promoted activation of STAT3 in the ovarian cancer cell line SKOV3, human umbilical vein endothelial cells (HUVECs), and PBMCs. We chose to examine the effect of HE4 on STAT3 activation and downstream regulation in SKOV3 cells since HE4 is predominantly known as an ovarian cancer biomarker^36^. Furthermore, we wanted to look at the effect of HE4 on signaling in HUVECs since these cells are frequently used to model angiogenesis *in vitro*. In all cell lines, treatment with a STAT3 inhibitor ablated HE4-mediated STAT3 activation (Fig. 3A). HIF1α protein expression was also found to be upregulated with HE4 treatment in HUVECs and PBMCs, and this upregulation was ablated with STAT3 inhibition (Fig. 3A). In order to test the effect of various doses of rHE4 on STAT3 activation, we treated HUVECs with 1, 5, 10, and 20 nM rHE4 and noted a similar degree of increase in phospho-STAT3 (Supplemental Fig. 1), suggesting there is a dose at which effects of HE4 become “saturated”.

Next, we measured levels of IL8 in the conditioned media of control and 20 nM rHE4-treated cells, and found an 18.00-fold increase in IL8 levels at 4 h (p = 0.0001), and a 35.06-fold increase at 24 h (p = 2.11 × 10^−10^) relative to control. Treatment with a STAT3 inhibitor reduced IL8 levels to 0.20-fold (p = 7.45 × 10^−6^) and 0.79-fold (3.64 × 10^−9^) relative to rHE4-treated at 4 h and 24 h, respectively (Fig. 3B). We also tested whether a lower concentration of HE4 would induce the same effects. Treatment of PBMC with 1 nM rHE4 caused a 5.32-fold increase in IL8 levels at 4 h (p = ns) and a 29.48-fold increase at 24 h (p = 0.0004) relative to control, indicating that 1 nM elicited similar effects as 20 nM rHE4 treatment. IL8 levels were likewise suppressed to 0.27-fold (p = 2.94 × 10^−5^) and 0.56-fold (p = 6.54 × 10^−6^) relative to rHE4-treated by addition of a STAT3 inhibitor at 4 h and 24 h, respectively (Fig. 3C). We also treated PBMC with rHE4 at earlier time points (1, 2, 4, 6 h) to narrow down the window of IL8 upregulation. A rise was observed as early as 2 h after treatment (11.77-fold, p=ns), showing that regulation of IL8 secretion by HE4 might begin to occur between 1 and 2 h. However, a more robust response occurs around 4 h (54.61-fold, p = 0.021), and continues to increase at 6 h (67.11-fold, 0.014)(Fig. 3D). These results highlight the importance of STAT3 signaling in HE4-mediated regulation of angiogenic factors in cells of the tumor microenvironment.

### HE4-mediated tube formation of endothelial cells is blocked by STAT3 inhibition

**Figure 4:**
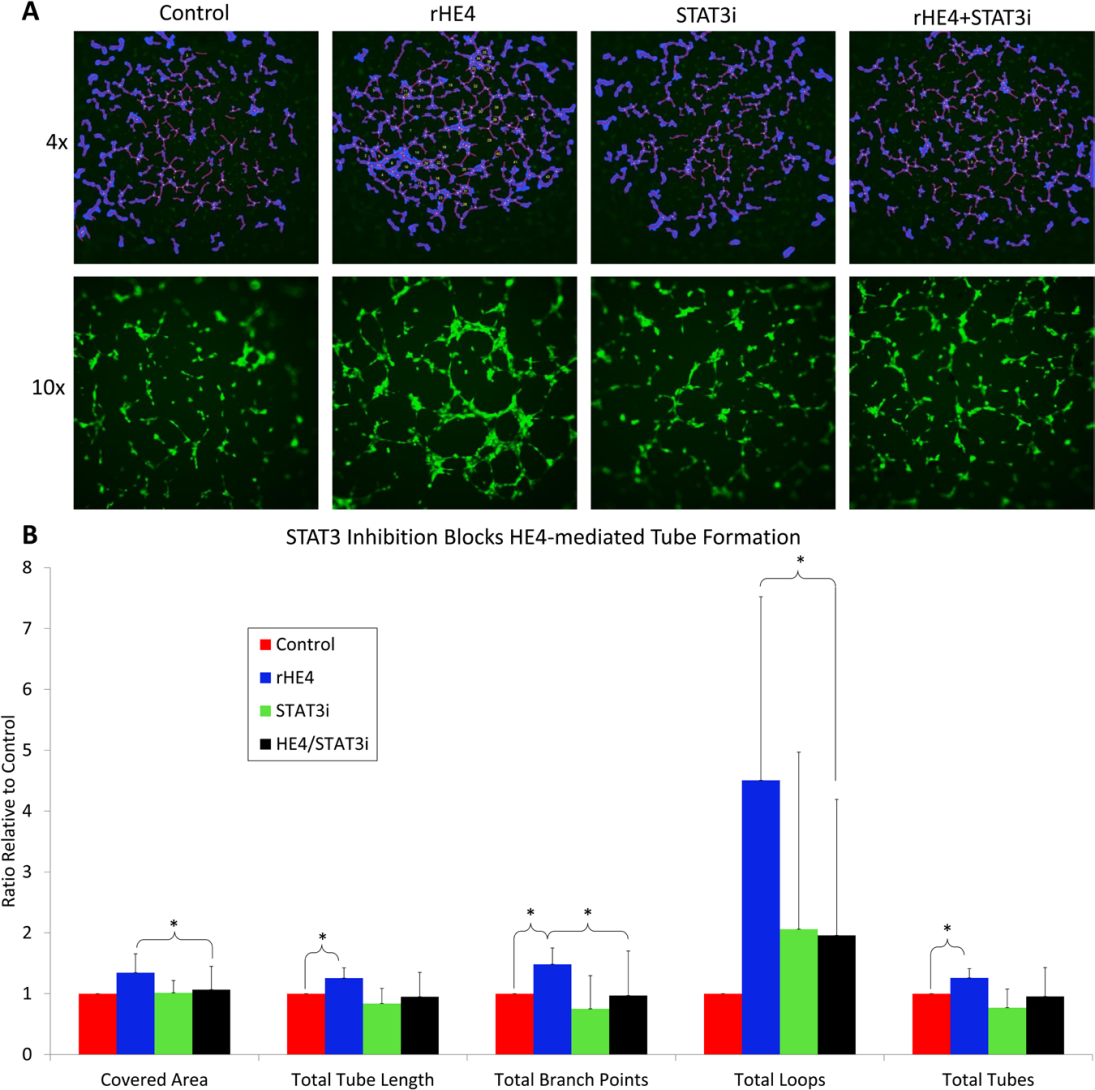
HE4-mediated tube formation of endothelial cells is blocked by STAT3 inhibition (**A**) rHE4 promotes tube formation of HUVECs, and STAT3 inhibition blocks tube formation in rHE4-treated cells. Top panel shows representative 4x images with tube features outlined in blue and tube numbers labeled. Bottom panel shows representative 10x images. **(B)** Quantification of tube formation from (**A**). Error bars represent standard deviation of at least three biological replicates. *p < 0.05, **p < 0.005.

To test the effect of HE4 on angiogenesis *in vitro*, we measured tube formation of HUVECs in response to 1 nM rHE4. HUVECs were plated on extracellular matrix, and tubes formed after 4–5 h. We treated the HUVECs with 1 nM rHE4 plus or minus 5 µM STAT3 inhibitor during tube formation. HE4-mediated increases were observed in covered area (1.34-fold, p = 0.064), total tube length (1.26-fold, p = 0.029), total branching points (1.48-fold, p = 0.018), total loops (4.5-fold, p = 0.057), and total tubes (1.26-fold, p = 0.021) with rHE4 treatment. No significant differences were detected in tube formation parameters between control and STAT3 inhibitor alone treated groups. However, tube formation was effectively reduced in the rHE4 plus STAT3 inhibitor group relative to rHE4-treated group, measured by covered area (0.78-fold, p = 0.011), total tube length (0.74-fold, p=ns), total branching points (0.62-fold, p = 0.021), total loops (0.30, p = 0.037), and total tubes (0.75, p=ns)(Fig. 4A,B).

### **HE4 is associated with IL8 levels, CD8** + **T cells, and microvascular density in EOC patient tissue**

**Figure 5:**
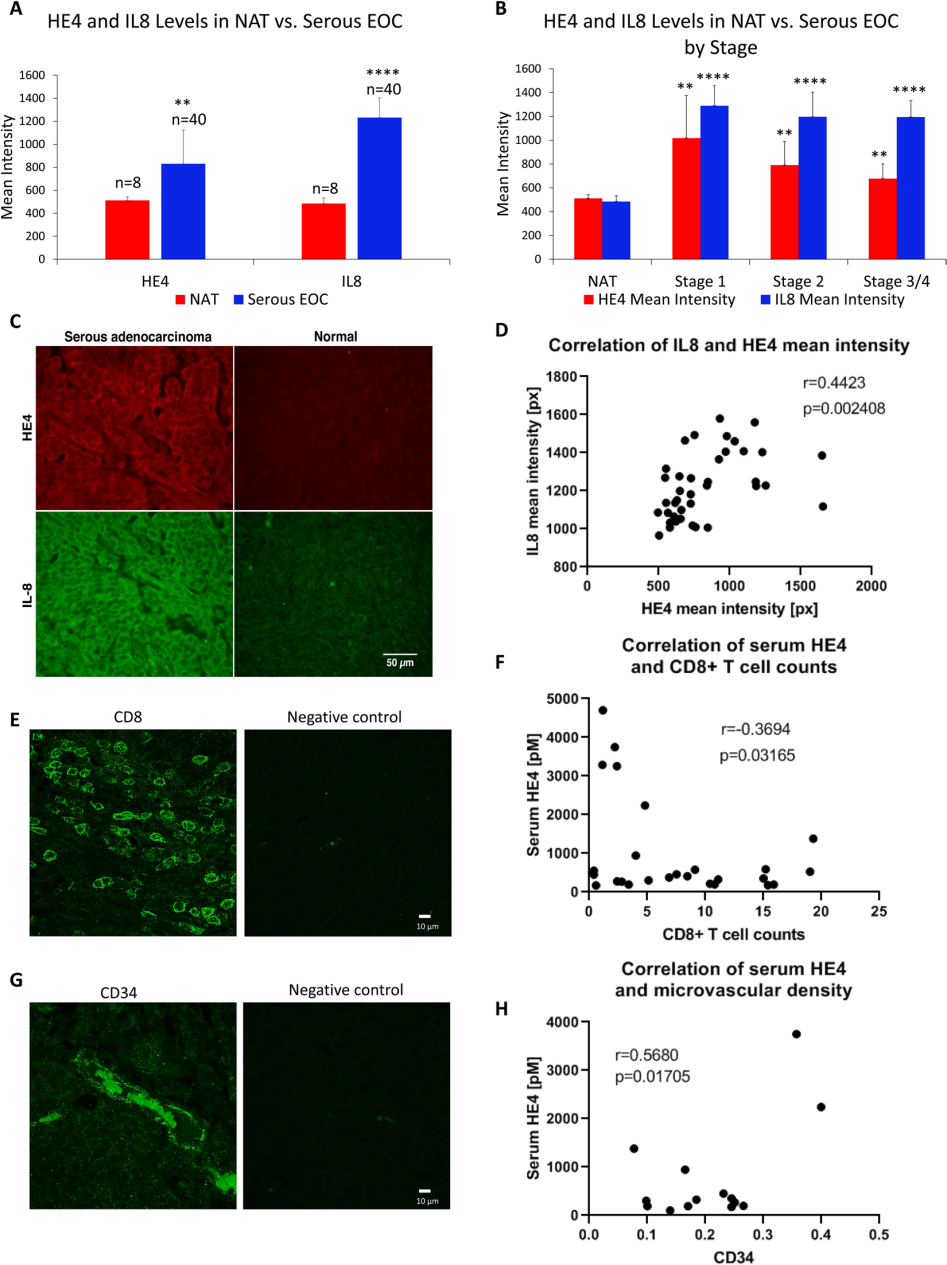
(**A**) HE4 and IL8 tissue levels are elevated in serous EOC compared to normal adjacent tissue (NAT) (**B**) HE4 and IL8 tissue levels are elevated in serous EOC at all stages compared to NAT. **(C)** Representative images of HE4 and IL8 staining in EOC tissue and NAT. **(D)** Tissue HE4 and IL8 levels (mean intensity) are positively correlated in serous EOC. **(E)** CD8 + T cells in serous EOC tissue. **(F)** Inverse correlation between HE4 serum levels and CD8 + T cell counts in patient EOC tissue (n = 26). **(G)** CD34 + microvascular staining in EOC tissue. **(H)** Correlation between HE4 serum levels and CD34 + area in patient EOC tissue (n = 14). **p < 0.005, ****p < 0.0005.

To begin to determine the clinical relevance of our findings, we performed fluorescent immunohistochemistry of IL8 and HE4 in serous adenocarcinoma EOC tissue (n = 40) and normal adjacent tissue (NAT; n = 8) in an EOC tissue microarray. As expected, HE4 levels and IL8 levels were both elevated in cancer tissue compared to NAT at all stages. Moreover, a significant positive correlation was noted between HE4 and IL8 mean intensity levels (Pearson r = 0.4423, p = 0.002)(Fig. 5A–D).

As a preliminary investigation into a potential relationship between HE4 and CD8 + cytotoxic T cells in EOC, we performed immunofluorescent analysis of total CD8 + T cells in serous EOC (n = 26)(Fig. 5E). Pearson correlation analysis indicated a significant inverse relationship between CD8 + T cells counts and HE4 serum levels (r = −0.3694; p = 0.032)(Fig. 5F). We also examined the relationship of serum HE4 levels with CD34 staining in EOC tissue (n = 14) as an indication of microvascular density. This analysis revealed a positive correlation between serum HE4 and CD34 + area (r = 0.5680; p = 0.017). All tumors examined were Stage III, grade 3 and naïve to chemotherapy, with the exception of one Stage IV tumor.

## Discussion

Collectively with previously published studies on the role of HE4 in ovarian cancer, a picture is emerging as to why HE4 is associated with poor prognosis in EOC patients. HE4 not only promotes aggressive characteristics of EOC cells, including enhanced proliferation, metastatic ability, and chemoresistance^22–31,37^, but it also has the ability to affect cells of the tumor microenvironment. The secretory nature of this protein allows it to have both intracellular, autocrine, and paracrine effects. Our results indicate that HE4 promotes activation of STAT3 signaling in multiple cell types, which occurs relatively rapidly after treatment. This result, combined with previously published studies showing that HE4 promotes ERK and FAK signaling^24,28,30,37^, shows that signaling is the likely major mode of action of HE4 regulation of multiple tumor characteristics.

The regulation of key angiogenic factors in immune cells apparently occurs via HE4-mediated STAT3 activation. Interestingly, STAT3 signaling has not been studied in ovarian cancer in the context of immune evasion. However, STAT3 is reported to promote immune suppression in multiple solid tumors^38–40^. The mechanisms of STAT3 immunosuppression in tumors is varied, since it is known to regulate a wide variety of genes^41^. One of its target genes is the well-known inhibitory T cell receptor ligand PD-L1^42^. Interestingly, HIF1α also transcriptionally regulates PD-L1^42^, and HIF1α in turn can bind to the HE4 gene promoter to upregulate its transcription^43^. While not examined in this study, we did note upregulation of PD-L1 by rHE4 in the qPCR arrays. Thus, these regulatory pathways may converge and overlap in various ways. The activation of STAT3 signaling by HE4 reveals that HE4 may induce many of its oncogenic effects in ovarian cancer via this major pathway; however, a complete picture of STAT3-dependent, HE4-mediated gene expression remains to be determined.

IL8 is a potent pro-angiogenic factor that plays a role in the pathogenesis of ovarian and several other cancers. *In vitro* tube formation assays and *in vivo* microvascular density measurements consistently reveal the pro-angiogenic nature of this cytokine^17,44–48^. IL8 concentrations in ovarian cancer ascites correlated with angiogenesis index when injected into mice^49^. Moreover, IL8 expression is associated with worse prognosis of high-grade serous ovarian cancer^50^. Likewise, HIF1α is a key transcription factor that promotes hypoxic adaptations including angiogenesis and metabolic changes in cancer^45,51^. For the first time, we have found a role for HE4 in promoting expression of these two genes in immune cells. This finding has implications for the immune response to ovarian cancer, by means of affecting angiogenesis and immune cell trafficking to the tumor. In agreement with this hypothesis, we found reduced CD8 + T cell infiltrate in tumors from patients with high serum HE4 levels.

Because of the known roles of IL8 and HIF1α in angiogenesis, and the observation that rHE4 increased STAT3 activation and HIF1α levels in HUVECs, we tested the effect of rHE4 on tube formation of HUVECs. We saw significant increases in measurements of tube formation with rHE4 treatment, and co-treatment with a low toxic dose of STAT3 inhibitor blocked tube formation in rHE4 treated cells. While this experiment demonstrated that HE4-mediated tube formation by HE4 was dependent on intact STAT3 signaling, other pathways could also be involved.

Although not examined here, another mechanism by which HE4 regulation of IL8 could promote angiogenesis and immune evasion is via neutrophil recruitment by IL8, which is a major function of this cytokine. Persistent neutrophil recruitment plays a role in carcinogenesis of *H. pylori* induced gastric cancer, and promotes angiogenesis and intravasation of fibrosarcoma and prostate cancer^52,53^. Furthermore, IL8 is secreted by tumor-associated macrophages (TAMs)^54^, so it would be interesting to explore the upregulation of IL8 by HE4 in various immune cell populations. While we know that this response occurs in a mixed population of immune cells, specific immune populations may also be responsive to HE4 induction of IL8 expression. These questions regarding complex interactions of the tumor microenvironment could be better answered using immune-competent mouse models of ovarian cancer.

Finally, it is important to note that we have not specifically examined the differences in HE4 effects depending upon its glycosylation status, which may be important. One study by Hua *et al*. found that aglycosylated HE4 is less inhibitory toward the substrates trypsin and elastase than the complex glycosylated form of HE4 that is secreted from mammalian cells^55^. While we have observed qualitatively that there may be a more robust effect resulting from treatment of ovarian cancer cells with conditioned media from HE4-overexpressing cells than with rHE4, we have not specifically determined whether quantitative differences exist between the two methods of HE4 exposure^30^. In this present study, we used rHE4 throughout, which we have shown to be partially glycosylated (Supplemental Fig. 2). Further studies are needed to get a complete understanding of how the degree of glycosylation affects results quantitatively, especially given that there appears to be a dose of HE4 at which the effects become saturated.

In conclusion, our study highlights a novel function of HE4 in regulating STAT3 signaling and pro-angiogenic factors in immune cells and tube formation of endothelial cells. These findings indicate that HE4 could have an important role in regulating both angiogenesis and suppression of immune cell infiltrate and responses. Collectively with its status as an ovarian cancer biomarker and previous studies showing roles for HE4 in proliferation, metastasis, chemoresistance, and immune suppression, HE4 could plausibly be targeted for therapeutic benefit and immunomodulatory effects.

## Methods

### Cell Culture and Treatments

SKOV3 cells were obtained from ATCC, and maintained at low passage. They were cultured in Dulbecco Modified Eagle Medium (DMEM) with 10% fetal bovine serum (FBS) and 1% penicillin/streptomycin (p/s), in a humidified incubator at 37 °C/5% CO_2_. Cells were plated at subconfluent density the day before treatment. Normal human PBMC from multiple female donors (ages 30–60; Precision for Medicine, Frederick, MD) were spun down from frozen stock and plated in RPMI media with 10% FBS and 1% pencillin/streptomycin. Pooled human umbilical vein endothelial cells (HUVECs) were provided from the Sharma and Shaw labs at Women & Infants Hospital, who obtained them from Lonza. They were cultured in EBM-Plus or EBM-2 complete medium (Lonza, CC-5036) on 0.1% gelatin-coated plates, up to passage 6.

SKOV3 cells and HUVECs were treated with recombinant HE4 (rHE4; My BioSource, MBS355616) at 1 nM (14 ng/mL) or 20 nM (280 ng/mL), for various time points, as described in the results. rHE4 is partially glycosylated (Supplemental Fig. 2), thus, the absolute bioactivity of a set concentration of rHE4 cannot be directly compared to levels *in vivo*. Furthermore, nanomolar concentration is estimated from the molecular weight of the aglycosylated protein. Therefore, in most cases, cells were treated with “excess” rHE4, as we observed similar results at both the lower and higher concentrations of rHE4.

In some cases, cells were treated alone or co-treated with STAT3 inhibitor VIII, 5, 15-DPP (Santa Cruz, sc-204305) at various concentrations (3.125 µM-50 µM) for indicated time points. The appropriate doses of the STAT3 inhibitor were determined by a dose response curve (0–100 µM), followed by MTS assay for SKOV3 and HUVECs. PBMC were treated with 1 and 20 nM rHE4 and/or 5 or 20 µM STAT3 inhbitor, and viability was determined by trypan blue live/dead counts. Various doses of STAT3 inhibitor were also tested for their ability to block HE4-mediated increases in phospho-STAT3 in HUVECs, SKOV3, and PBMCs. The doses that effectively inhibited STAT3 activation while minimizing effects on viability were used throughout this study. (Supplemental Fig. 3).

### Quantitative PCR Array

Normal human PBMCs were plated with or without 20 nM rHE4 for 6 h. Cells were spun down, washed with PBS, and lysed in Trizol for RNA isolation using high salt and lithium chloride precipitation. Total RNA was quantified by Nanodrop and reverse transcribed using the RT2 First Strand cDNA Kit (Qiagen, 330401). The cDNA was then used in an RT2 Profiler Cancer Inflammation and Immunity Crosstalk human array (Qiagen, PAHS-181Z), according to the manufacturer’s instructions. The plates were run on an ABI 7500 qPCR machine, with 10 min at 95 °C, and forty cycles of 15 s at 95 °C and 1 min at 60 °C. An automated baseline was used, with thresholds manually adjusted to remain constant across all plates. Data was analyzed using the GeneGlobe Data Analysis Center at Qiagen.com. GAPDH was used as the normalization gene. Calculations of relative expression levels were performed using the using the ΔΔCt method. All samples passed quality control standards (array reproducibility, RT efficiency, and genomic DNA contamination). The array was repeated two independent times, with each experiment pooling three control and three rHE4-treated replicates.

### Quantitative PCR

Quantitative PCR was performed as previously described^30^. Validated primers for *CSF3*, *IL8, HIF1A* and *STAT3* were purchased from realtimeprimers.com. PrimePCR *VEGFA* assay was purchased from BioRad. All figures represent at least three biological replicates with three technical replicates each. Custom primer sequences (Invitrogen) are as follows:

18 s rRNA (F) – CCG CGG TTC TAT TTT GTT GG

18 s rRNA (R) – GGC GCT CCC TCT TAA TCA TG

### Western Blot

Western blot was performed as previously described^30^. GAPDH was used as a loading control. Uncropped blots can be seen in Supplemental Fig. 4. Antibodies and dilutions used are as follows:

GAPDH (Cell Signaling, 2118, 1:2000)

Phospho-STAT3 (Cell Signaling, 9131, 1:1000)

STAT3 (Cell Signaling, 30835, 1:1000)

HE4 (Origene, TA307787, 1:2000)

HIF1A (Novus, NB-100–134SS, 1:200)

### MTS Viability Assay

For dose curve experiments, SKOV3 and HUVECs were plated at ~5,000 cells/well in 96-well plates. Cells were treated with varying doses of STAT3 inhibitor for 24 h, and MTS assay was performed. 10 µl CellTiter 96® Aqueous One Solution (Promega, G3580) was added to each well, allowed to incubate for 2–4 h in a humidified chamber at 37 °C/5% CO2. Absorbance was read at 492 nm. Dose curve experiments were performed as a single experiment with three or more biological replicates.

### Angiogenesis Assay

To measure tube formation of HUVECs, HUVECs were labeled with 2 nM cell tracker green (Thermo Fisher, C7025) for 30 m. Meanwhile, 96-well plates were coated with 50 µl reduced growth factor Geltrex (Thermo Fisher, A1413202) for 1 h at 37 °C. Next, 10,000 HUVEC/well were added, with or without rHE4 (1 nM) and/or STAT3 inhibitor VIII (5 µM). Cells were observed for tube formation, then stained and imaged after 4–5 h. Fluorescent images were analyzed using the Wimasis Wimtube program. The experiment was repeated in three separate experiments with ≥3 biological replicates per experiment.

### Ovarian cancer tissue samples

An ovarian cancer microarray was obtained from US Biomax (OV802a). Residual EOC tissue was obtained from the Pathology Department at Women & Infants Hospital under Institutional Review Board approval. Serum HE4 levels in patients whose tissue was stained for CD8 + T cell infiltration or CD34 were obtained from patient medical records (HE4 levels in these patients were determined in the Special Testing Department during routine medical care, and values were obtained from chart review under Institutional Review Board approval). All experiments were approved and performed in accordance with relevant guidelines and regulations of the Women and Infants Hospital Institutional Review Board. Tissue was obtained with a waiver of informed consent and HIPAA authorization.

### Immunofluorescence

HUVECs were imaged at 4x and 10x on a Nikon Ti-E using the widefield 488 laser. For 4x images that were used for analysis, one image per well was taken, which included a majority of the well.

CD8 + T cells were stained in human serous EOC tissue (n = 26) using an anti-CD8 antibody (Origene, TA802079, 1:50) and DyLight 488 secondary. Immunohistochemistry was performed as previously described^56^. Cell counts per 40x field were performed in ten random fields and averaged.

Microvascular density was determined in serous EOC tissue (n = 14) by staining for CD34 (Novus, NBP2-67399). Ten fields with the most CD34 positive staining were identified and acquired per sample. A Nikon E800 microscope, (Nikon Inc. Mellville, NY), with a Plan Apo 40x objective and a Spot RT3 camera (Diagnostic Instruments, Sterling Heights MI) was used to acquire all images. Each wavelength was acquired separately and an RGB image was created. Image processing and analysis was performed using iVision image analysis software (BioVision Technologies, version 4.0.10, Exton, PA). Positive staining was defined through intensity thresholding of the green channel. Images were calibrated so that results are expressed as CD34 positive area per total square micrometers.

Confocal images for CD8 and CD34 were acquired with a Nikon C1si confocal (Nikon Inc. Mellville NY.) using diode laser 488. Serial optical sections were obtained with EZ-C1 computer software (Nikon Inc. Mellville, NY). Z series sections were collected with a 40x Plan Apo objective and a scan zoom of 2, images were collected every 0.30 µm. Deconvolution and projections were performed with Elements software (Nikon Inc. Mellville NY).

Immunofluorescent staining of the ovarian cancer microarray was performed as previously described^32^, using antibodies for HE4 (Santa Cruz, sc-293473) and IL8 (Santa Cruz, sc-8427). Two to three fields/sample were randomly selected based on DAPI staining, and minimum, mean, and maximum gray values (pixels) were determined for each field. Normal adjacent tissues were used to set the threshold for positive staining. All T cell counts, analysis, and imaging except for the angiogenesis assay were performed at the Rhode Island Hospital Digital Imaging Core Facility.

### ELISA

IL8 ELISA was performed with a human IL8 ELISA kit (Invitrogen, KHC0081), according to the manufacturer’s instructions. All ELISA experiments were repeated in three independent experiments with 1–3 biological replicates each.

### Statistics

Pearson correlation was performed in Prism to determine r-values and p-values for IL8, CD8, and CD34 correlations with HE4. For quantitative PCR and MTS assay, p-values were determined using unpaired, one-tailed student t-test. Differences were considered statistically significant when p < 0.05. Biological replicates ≥3 for all experiments showing statistical significance.

## Supplementary information

Supplemental Information.

Supplemental Dataset.
