# Supplementary material for: The biomarker HE4 (WFDC2) promotes a pro-angiogenic and immunosuppressive tumor microenvironment via regulation of STAT3 target genes
Source: Sci Rep. 2020 May 22;10:8558. doi: 10.1038/s41598-020-65353-x (PMC7244765; doi:10.1038/s41598-020-65353-x)
Supplement: Supplementary file 1 — Supplemental Information. [file 41598_2020_65353_MOESM1_ESM.pdf]

The biomarker HE4 (WFDC2) promotes a pro-angiogenic and immunosuppressive tumor microenvironment via regulation of STAT3 target genes

Authors: Nicole E James[1], Jenna B Emerson[1,2], Ashley D Borgstadt[1,2], Lindsey Beffa[1,2], Matthew T Oliver[1,2], Virginia Hovanesian[3], Anze Uhr[4], Rakesh K Singh[5], Rachael Rowswell-Turner[5], Paul A DiSilvestro[1,2], Joyce Ou[2,6], Richard G Moore[5], Jennifer R Ribeiro[1,2*]

[1] Women and Infants Hospital, Department of Obstetrics and Gynecology, Program in Women's Oncology, Providence, RI
[2] Warren-Alpert Medical School of Brown University, Providence, RI
[3] Rhode Island Hospital, Digital Imaging and Analysis Core Facility, Providence, RI
[4] Northwell Health Physician Partners Gynecologic Oncology, Brightwaters, NY
[5] University of Rochester Medical Center, Rochester, NY
[6] Women and Infants Hospital, Department of Pathology, Providence, RI

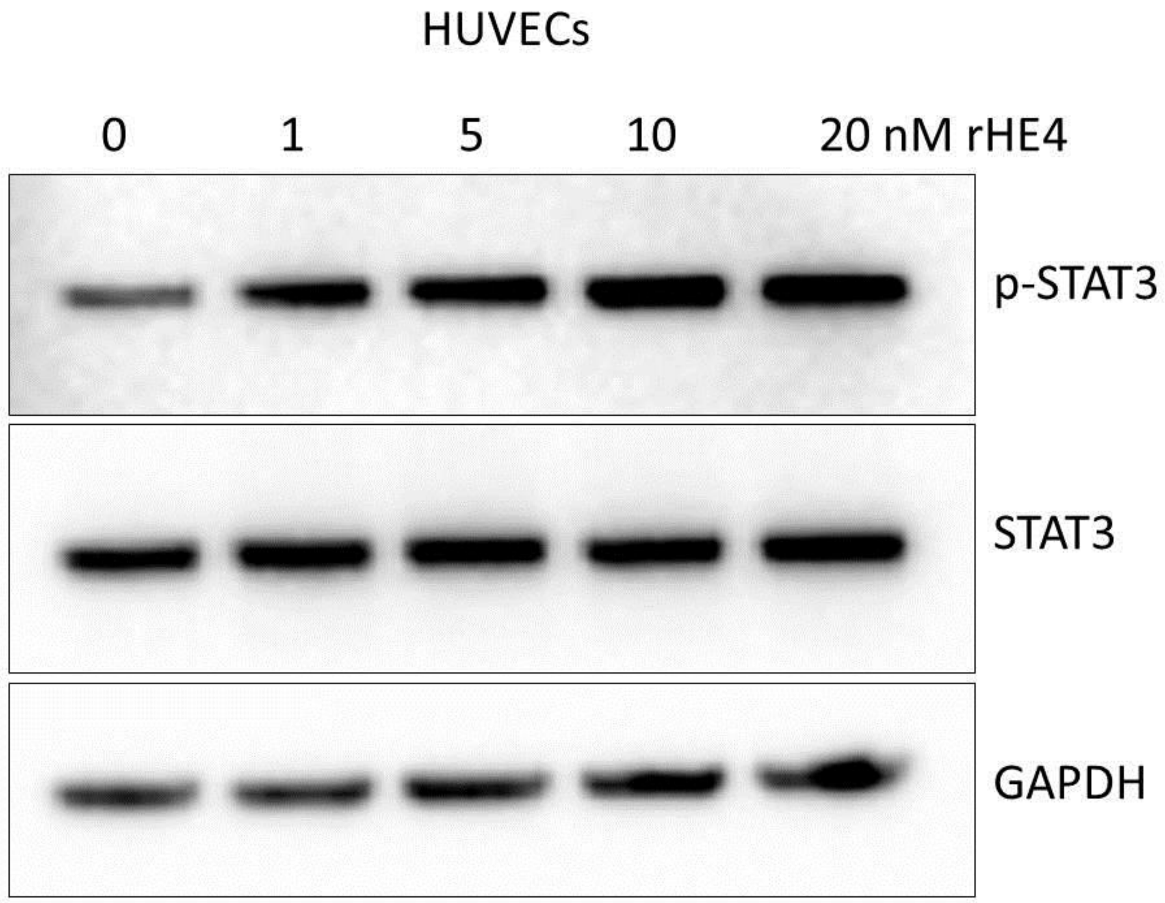

Supplemental Figure 1 Various doses of rHE4 activate STAT3 signaling in HUVECs. HUVECs were treated with 1, 5, 10, and 20 nM rHE4 for 24 h, and levels of phospho-STAT3 were evaluated by western blot.

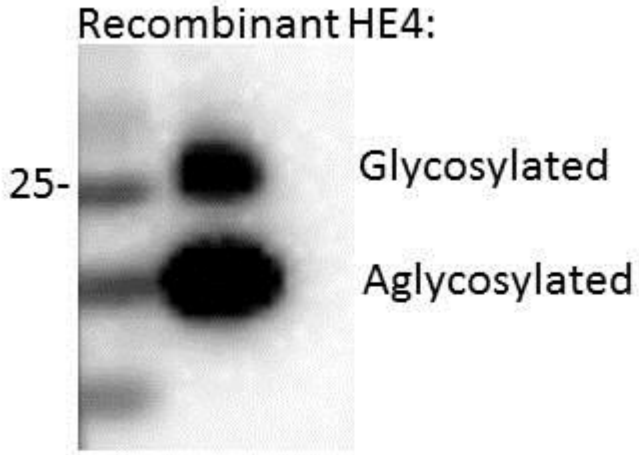

Supplemental Figure 2 Recombinant HE4 is partially glycosylated Western blot showing the presence of glycosylated HE4 (top band) and aglycosylated HE4 (bottom band) in recombinant HE4 protein prep.

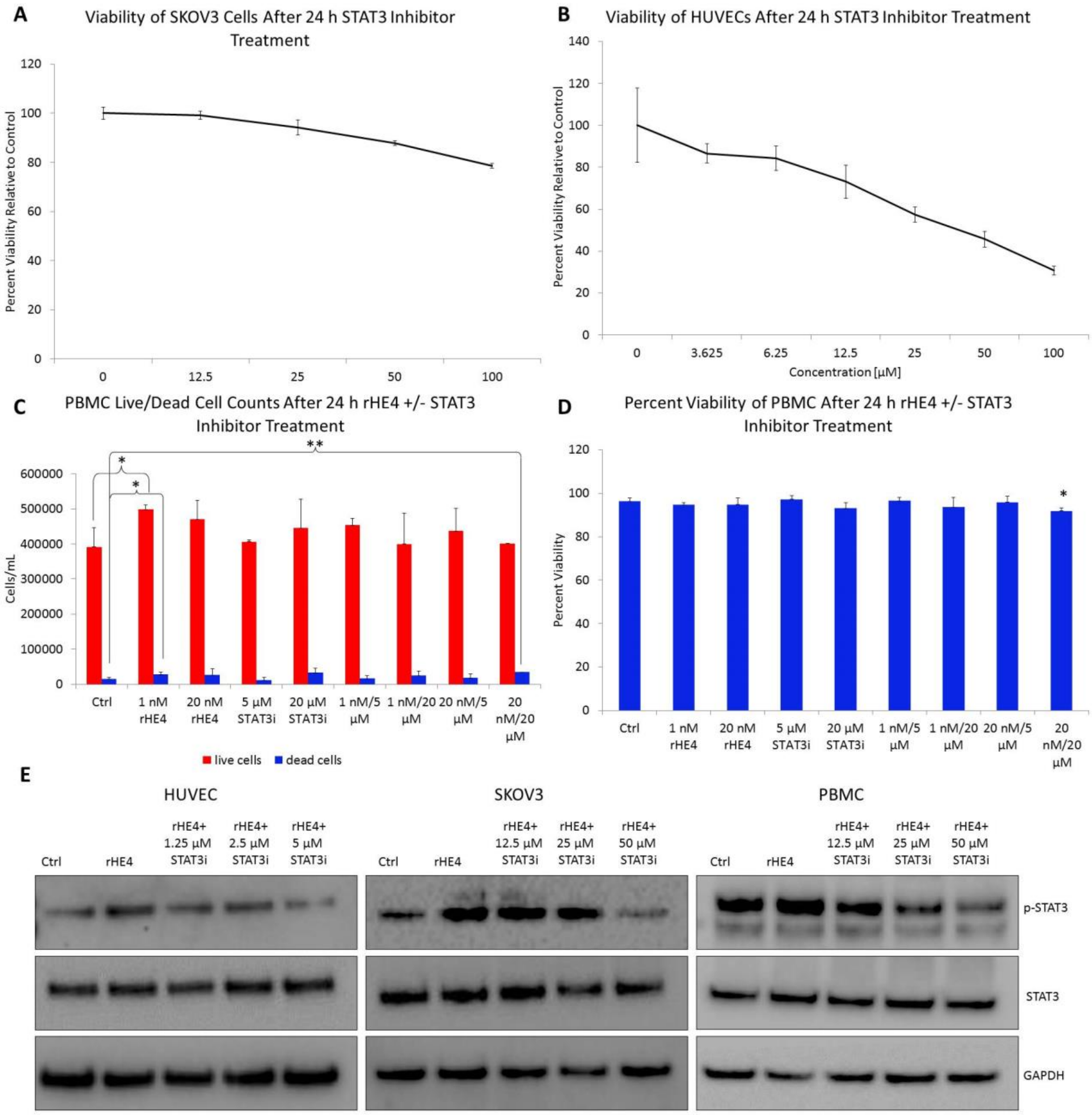

Supplemental Figure 3 Cell viability with STAT3 inhibitor treatment (A) Percent viability of SKOV3 cells treated with increasing doses of STAT3 inhibitor for 24 h. **(B)** Percent viability of HUVECs treated with increasing doses of STAT3 inhibitor for 24 h. **(C)** Live/dead cell counts of PBMCs treated with indicated doses of rHE4 and/or STAT3 inhibitor for 24 h. **(D)** Percent viability of PBMCs treated with indicated doses of rHE4 and/or STAT3 inhibitor for 24 h. Error bars represent standard deviation. *p<0.05 **(E)** Western blot of HUVECs, SKOV3, and PBMCs showing phospho-STAT3 and STAT3 levels in response to 24 h treatment with rHE4 with increasing doses of STAT3 inhibitor. GAPDH was used as a loading control.

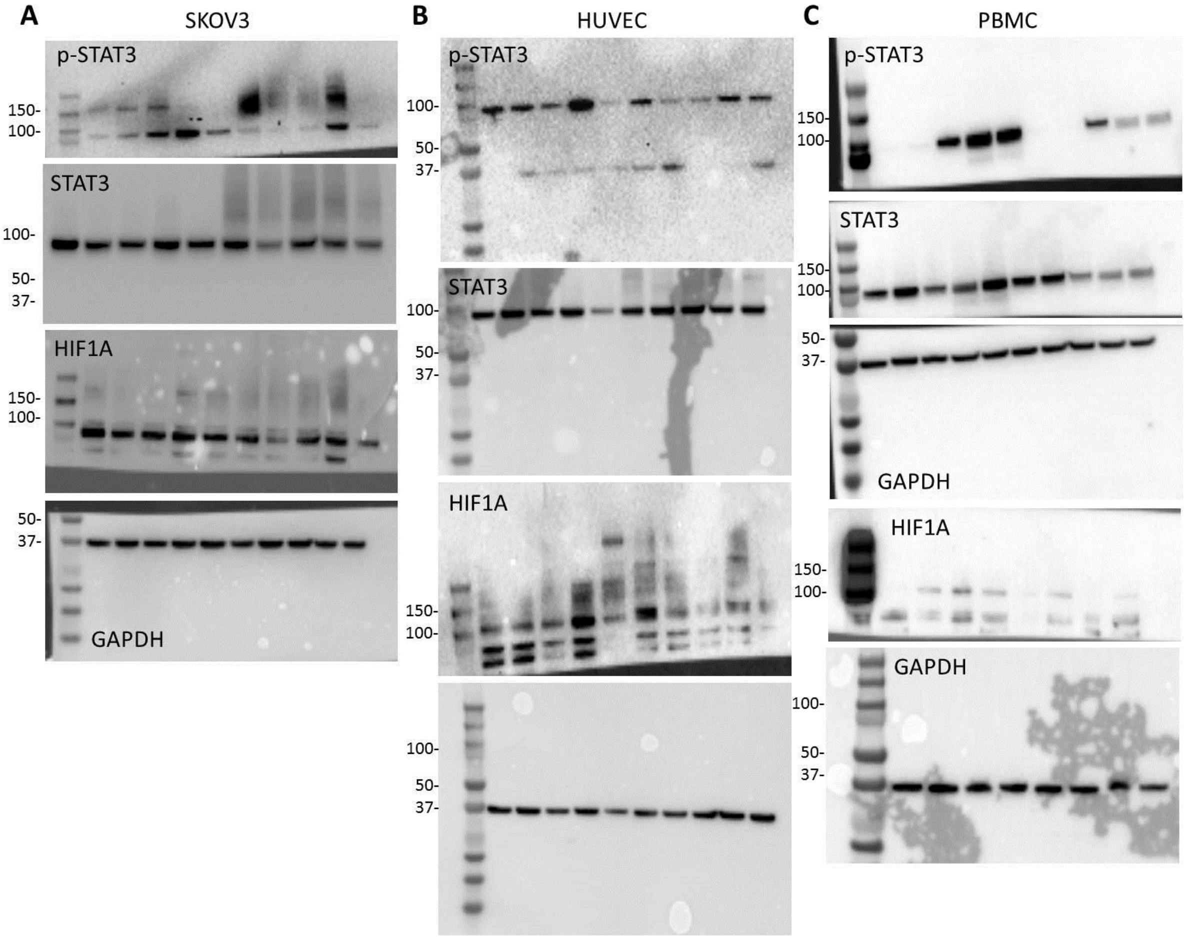

Supplemental Figure 4 Uncropped western blots (A) SKOV3 western blots. **(B)** HUVEC western blots. **(C)** PBMC western blots.
